# TNFα induces Caspase-3 activity in hematopoietic progenitor cells CD34+, CD33+, and CD41 + of myelodysplastic syndromes

**DOI:** 10.1186/s12860-023-00495-0

**Published:** 2023-11-21

**Authors:** Anggraini Iriani, Andhika Rachman, Rahayuningsih D. Setiabudy, Siti B. Kresno, Aru W. Sudoyo, Mansyur Arief, Alida R. Harahap, Marsya Kaila Fatina

**Affiliations:** 1 Department of Clinical Pathology, Faculty of Medicine, Yarsi University - Yarsi Hospital, Jl. Letjen Suprapto Kav 13, Cempaka Putih, Jakarta, 10510 Indonesia; 2 Division of Hematology and Medical Oncology, Department of Internal Medicine, Faculty of Medicine, Universitas Indonesia - Dr. Cipto Mangunkusumo General Hospital, Jakarta, Indonesia; 3 Department of Clinical Pathology, University of Indonesia, Jakarta, Indonesia; 4 Department of Clinical Pathology, Hasanuddin University, Makasar, Indonesia; 5 Faculty of Medicine, Andalas University, Padang, Indonesia

**Keywords:** Myelodysplastic syndrome, TNFα, caspase-3, CD34+, CD33+, CD41+

## Abstract

**Background:**

Cytopenia is the primary feature of Myelodysplastic Syndrome, even in the presence of hypercellular bone marrow. TNFα is recognized as both a proinflammatory, and proapoptotic cytokine with a well established role in promoting apoptosis in MDS. Therefore, TNFα has the potential to be a valuable biomarker for predicting the progression of cytopenia in MDS. This study aims to establish the role of TNFα exposure in triggering apoptosis through caspase-3 activity in CD34+, CD33+, and CD41 + cells in MDS.

**Methods:**

This study is an in vitro comparative experimental research. Bone marrow mononuclear cells were isolated as the source of hematopoietic progenitor cells. Subsequently, CD34+, CD33+, and CD41 + cells were exposed to rhTNFα, and the caspase-3 activity was measured using flowcytometry.

**Results:**

In MDS CD33 + and CD41 + caspase-3 activity of rhTNFα exposed cells was significantly higher than without exposed cells. The opposite result was found in CD34 + cells, where the caspase-3 activity without rhTNFα exposed cells was significantly higher than rhTNFα exposed cells.

**Conclusion:**

rhTNFα exposure led to an elevation in caspase-3 activity in MDS progenitor cells, especially in those that had differentiated into myeloid cell CD33 + and megakaryocyte cell CD41+, as opposed to the early progenitor cells CD34+.

**Supplementary Information:**

The online version contains supplementary material available at 10.1186/s12860-023-00495-0.

## Introduction

Myelodysplastic syndrome (MDS) is a heterogeneous clonal stem cell disorder characterized by abnormalities in the differentiation and proliferation of hematopoietic precursors which leads to peripheral blood cytopenia, bone marrow hypercellularity, ineffective hematopoiesis, and dysplastic changes in myeloid, erythroid, and megakaryocyte lineages [1–4]. Cytopenia is the prominent feature of MDS amid the hypercellularity in the bone marrow [5, 6]. Some experts postulate that ineffective hematopoiesis in MDS may be caused by the increase in hematopoietic cell apoptosis, coupled with heightened intra-medullary proliferation or self-renewal capacity [7]. The bone marrow’s microenvironment is a highly intricate structure that plays a crucial role in regulating the proliferation, differentiation, and self-renewal processes of progenitor cells by providing essential factors such as nutrients, growth factors, and cytokines. However, in MDS, the bone marrow microenvironment undergoes changes that involve interactions between various cell types and between cells and cytokines, which contribute to the pathogenesis of the MDS [8, 9].

There are several hypotheses explaining the heightened occurrence of apoptosis. Firstly, increased apoptosis serves as a mechanism through which hematopoiesis cells dispose of undesired cells. When bone marrow progenitor cells come across chronic peripheral cytopenia, the body’s feedback mechanism triggers the production of cytokines to facilitate the formation of a new cell. Secondly, the elevation in apoptosis can be attributed to clonal abnormalities in MDS, implying that apoptosis is not solely an outcome but is intricately linked to the underlying cause or mechanism of abnormal biology in MDS clones [10, 11].

The TNFα gene is located on chromosome 6p21.3 and spans a length of 3 kb, encompassing four exons. The final exon is responsible for encoding approximately 80% of the secreted TNFα. TNFα itself is a type 2 transmembrane protein, composed of 212 amino acids and form a homotrimer structure. Various cell types, including macrophages, monocytes, T cells, B cells, NK cells, and Kupffer cells, produce TNFα. Its production is induced in response to cytokine signals, such as GMCSF, IL-1, IL-2, IFNγ, and immune complexes [12].

Apoptotic activity is assumed to be the primary mechanism underlying ineffective hematopoiesis, leading to cytopenia in the peripheral blood of MDS patients [8, 13]. In these patients, there is a known elevation in the level of TNFα, a cytokine that triggers apoptosis via the extrinsic pathway [14]. TNFα interacts with the TNF receptor initiating the activation of the caspase cascade. Caspase is an intracellular proteolytic enzyme responsible for triggering apoptosis [15]. Specifically, activation of caspase-3 triggers a cell to initiate apoptosis. The activity of this enzyme is used as a marker for apoptotic signaling in cells. However, the exact location within MDS progenitor cells where this TNFα-induced apoptotic process occurs remains unknown, whether it takes place in the early progenitor cells (CD34 +) or in the differentiated myeloid progenitor cells (CD33 +) and megakaryocyte progenitor cells (CD41+) [10, 11].

This study aims to demonstrate the impact of TNFα exposure on inducing apoptotic activity in hematopoietic progenitor cells, specifically CD34+, CD33+, and CD41 + cells in MDS. We aim to show that this exposure leads to an increase in caspase-3 activity particularly in cells that have undergone differentiation into myeloid, and megakaryocyte lineages.

## Materials and methods

This study employed a comparative in vitro experimental design involving the isolation of bone marrow mononuclear cells (BMMC) from 15 MDS bone marrow aspirates. Diagnosis of MDS was established based on clinical examination, complete hematological examination, peripheral blood and bone marrow morphology analysis [15], as well as cytogenetic testing according to the International System for Human Cytogenomic Nomenclature (ISCN) recommendations to align with the 2016 WHO diagnostic criteria [2]. The samples used consisted of CD34+, CD33+, and CD41 + cells that were isolated from the BMMC obtained from MDS patients. As a control group, we isolated BMMC from 8 lymphoma bone marrow aspirates, because it was not feasible to obtain bone marrow aspirates from healthy individuals, and lymphoma typically exhibits abnormalities in non-myeloid lineages, Table 1.

**Table 1:** Characteristic of MDS bone marrow aspirate

Bone marrow Aspirate	Bone Marrow Morphology	IPSS-R (score)
MDS 1	MDS-SLD	High (5)
MDS 2	MDS-SLD	Intermediate (4,5)
MDS 3	MDS-SLD	Very high (7)
MDS 4	MDS-SLD	NA
MDS 5	MDS-MLD	High (4,5)
MDS 6	MDS-MLD	Low (2,5)
MDS 7	MDS-MLD	Intermediate (4,5)
MDS 8	MDS-MLD	Very low (1)
MDS 9	MDS-MLD	NA
MDS 10	MDS-MLD	Intermediate (3,5)
MDS 11	MDS-MLD	NA
MDS 12	MDS-EB-1	Very high (7,5)
MDS 13	MDS-EB-1	Intermediate (4)
MDS 14	MDS-EB-1	Intermediate (4,5)
MDS 15	MDS-EB-1	High (6)

*MDS *Myelodysplastic syndrome, *MDS-SLD *Single lineage dysplasia, *MDS-MLD *Multilineage dysplasia, *MDS-EB-1 *Excess blast 1, *NA  *Not applicable, *IPSS-R* international prognostic scoring system revised

### BMMC isolation

Isolation of BMMC was performed using density gradient centrifugation with Ficoll-Paque medium (GE Healthcare™, Sweden). A cell density of 300,000 BMMC in 300 µL of StemMACS HSC media (Miltenyi Biotec™, USA) was used in each exposure. Cells were prepared in separate tubes, with some exposed to 48ng/100mL recombinant human TNFα / rhTNFα (R&D *systems*™, USA) followed by incubation at 37^O^C with 5% CO_2_ for 18 h. The remaining cells received the same treatment without exposure to TNFα,

### Caspase 3 activity measurement

The BMMC from both the MDS and control groups underwent identical treatments, involving exposure to TNFα and no exposure. Each group was examined for caspase-3 activity, both with and without exposure to the TNFα. Then, the delta (Δ ) caspase-3 activity was measured, representing the difference between caspase-3 activity in BMMC exposed to rhTNFα and those without exposure. The BMMC were then labeled with specific fluorochrome antibodies; (CD34 + with APC, CD33 + with PE, and CD41 + with PE); and a fluorochrome FITC label was applied to assess caspase-3 activity (BD™, USA). The fluorescence intensity was quantified using the BD FACSCalibur flowcytometry, Table 2.

**Table 2:** Caspase-3 examination panel

Tube^a^	Treatment	Fluorochrome antibody
1	Without exposure	Negative control
2	Cisplatin 100 µg/(10 µL)	Positive control
3	Without exposure	CD34 APC, CD40 PE, CD154 FITC
4	Without exposure	CD34 APC, Caspase-3 FITC
5	rhTNFα 48 ng	CD34 APC, Caspase-3 FITC
6	Without exposure	CD33 PE, Caspase-3 FITC
7	rhTNFα 48 ng/well	CD34 APC, CD33 PE, Caspase-3 FITC
8	Without exposure	CD41 PE, Caspase-3 FITC
9	rhTNFα 48 ng/well	CD34 APC, CD41a PE, Caspase-3 FITC
10	Without exposure	CD73 PE, Caspase-3 FITC
11	rhTNFα 48 ng/well	CD34 APC, CD73 PE, Caspase-3 FITC

^a^Each tube contains BMMC with density of 300,000 cells/300µL media

**Fig. 1:**
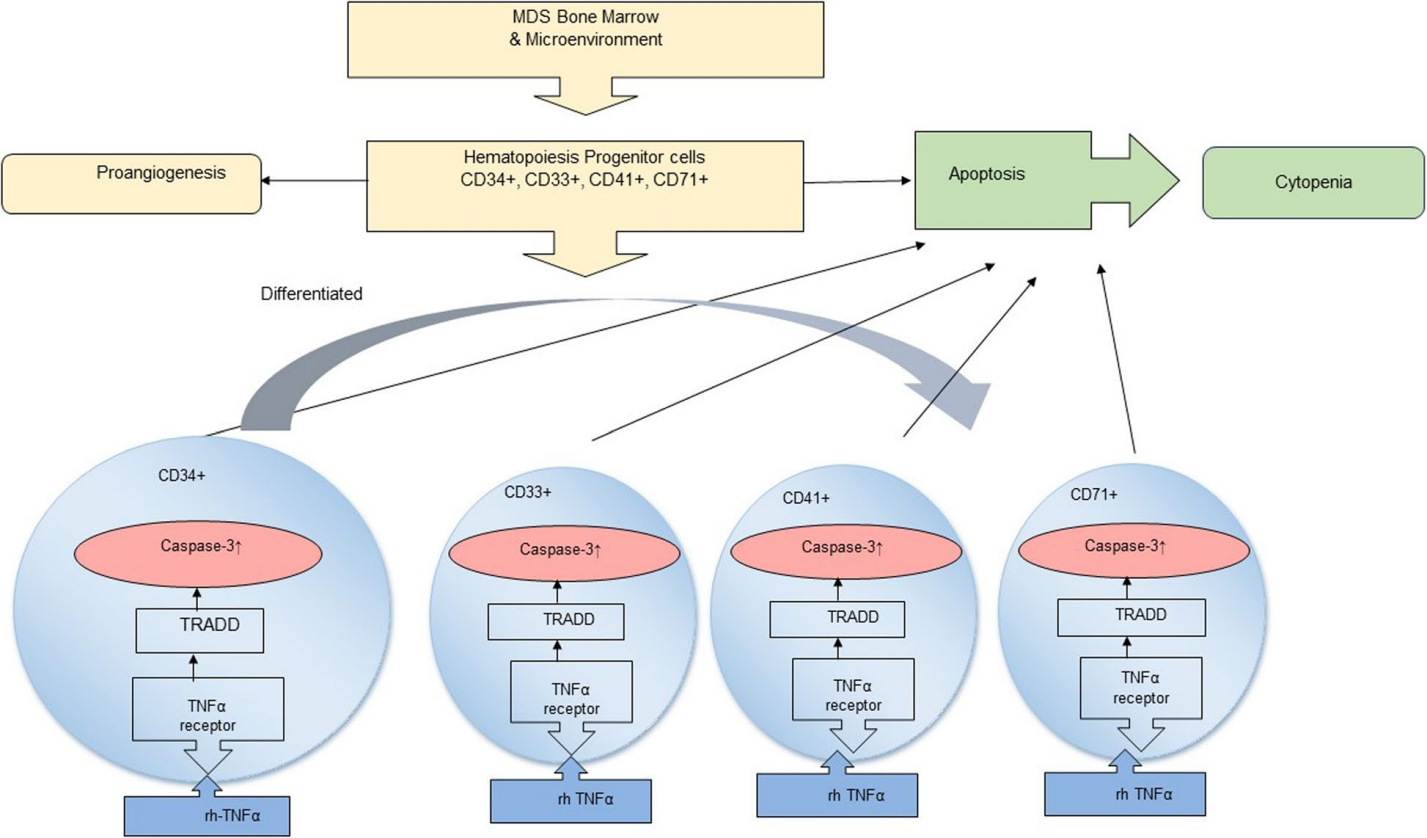
The idea is that exposure of rhTNFα to the BMMC of MDS, CD34+, CD33+, and CD41 + would induce apoptosis through extrinsic pathway via TRADD and activate cascade caspase to increase caspase-3 activity

The conceptual framework of this study is the BMMC from MDS patients, consist of CD34+, CD33+, and CD41 + in microenvironment of bone marrow there are conditions that support proapoptotic. This study tries to look at these in vivo condition in vitro. The apoptosis process which is though to be mainly induced by TNFα was tested by exposing rhTNFα to CD34+, CD33+, and CD41+, to find out in which progenitor lineage the apoptosis process occurs more dominantly by examining caspase-3 activity, Fig. 1.

### Flowcytometry analysis

Measurement of active caspase-3 was conducted using flow cytometry, which involves passing cells (events) through a detector. A number of cells were examined and analyzed on a scatter diagram. The x-axis represented the intensity of FITC against caspase-3 in the cells, while the y-axis described the fluorescence intensity against cell markers (CD34, CD33, CD41). Cells displaying a fluorescent pattern were identified and compared with the entire cell population, after which the percentage was calculated.

### Statistical analysis

To analyze the data for differences between paired groups (those without exposure and those exposed to rhTNFα) a paired t-test was employed, whereas, for unpaired groups (comparing the MDS group to the control group) an independent t-test was used.

The comparison of ∆ caspase-3 activity between the CD34+, CD33+, and CD41 + cell groups in MDS and the corresponding control cell groups was conducted. Both groups under conditions without exposure and after exposure to rhTNFα. A one-way ANOVA test was performed for more than two unpaired groups. If the results of the One-Way ANOVA test indicated a *p*-value of < 0.001. Therefore, it can be concluded that there were at least two distinct variations in the caspase-3 activity mean among the progenitor cell groups, which were significantly different in both the MDS and control groups.

## Results

**Fig. 2:**
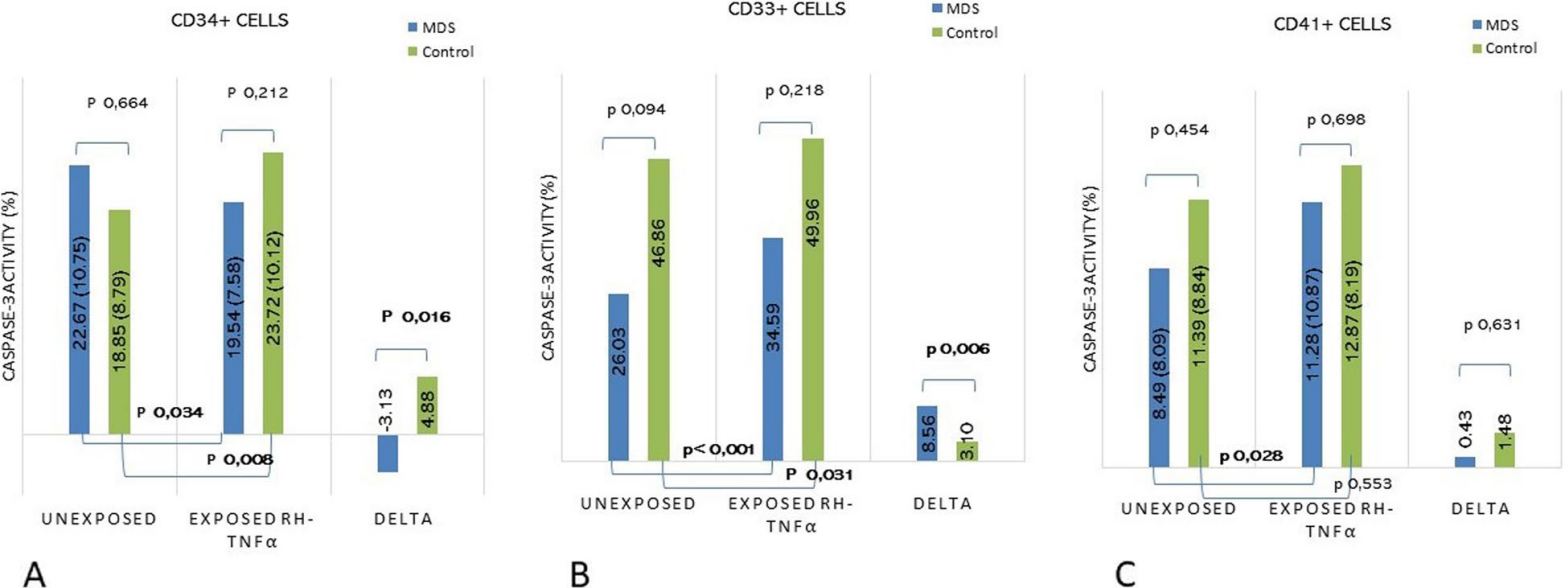
The caspase-3 activity in MDS and control BMMC unexposed and exposed to rhTNFα. **A** Caspase activity in CD34 + cells, **B** Caspase-3 activity in CD33+, **C** Caspase activity in CD41 + cells.  Data presented in mean (SD). Blue box: MDS group; green box: control group.  Left side: the group of cells unexposed to rhTNFα; middle side: the group of cells exposed to rhTNFα; right side: delta value. Delta formula: caspase-3 activity after exposure rhTNFα subtracted by caspase-3 activity before exposure

The mean of Δ activity caspase-3 in CD34 + between MDS and control groups showed that there was a significant difference with *p*-value 0.016. The paired t-test to analyze caspase-3 activity in CD34 + MDS without exposure and those exposed to rhTNFα showed a significant difference with *p*-value 0.034, as well as in control with *p*-value 0.008. In the MDS group the mean of caspase-3 activity in CD34 + without exposure (22.67%) was higher than in CD34 + exposed to rhTNFα (19.53%), with a negative Δ activity caspase-3. In control group the mean caspase-3 activity in CD34 + exposed to rhTNFα (23.72%) was higher than without exposure (18.84%), Fig. 2. This shows that CD34 + has weird behavior because even without exposure to rhTNFα the caspase-3 activity tends to be higher.

**Fig. 3:**
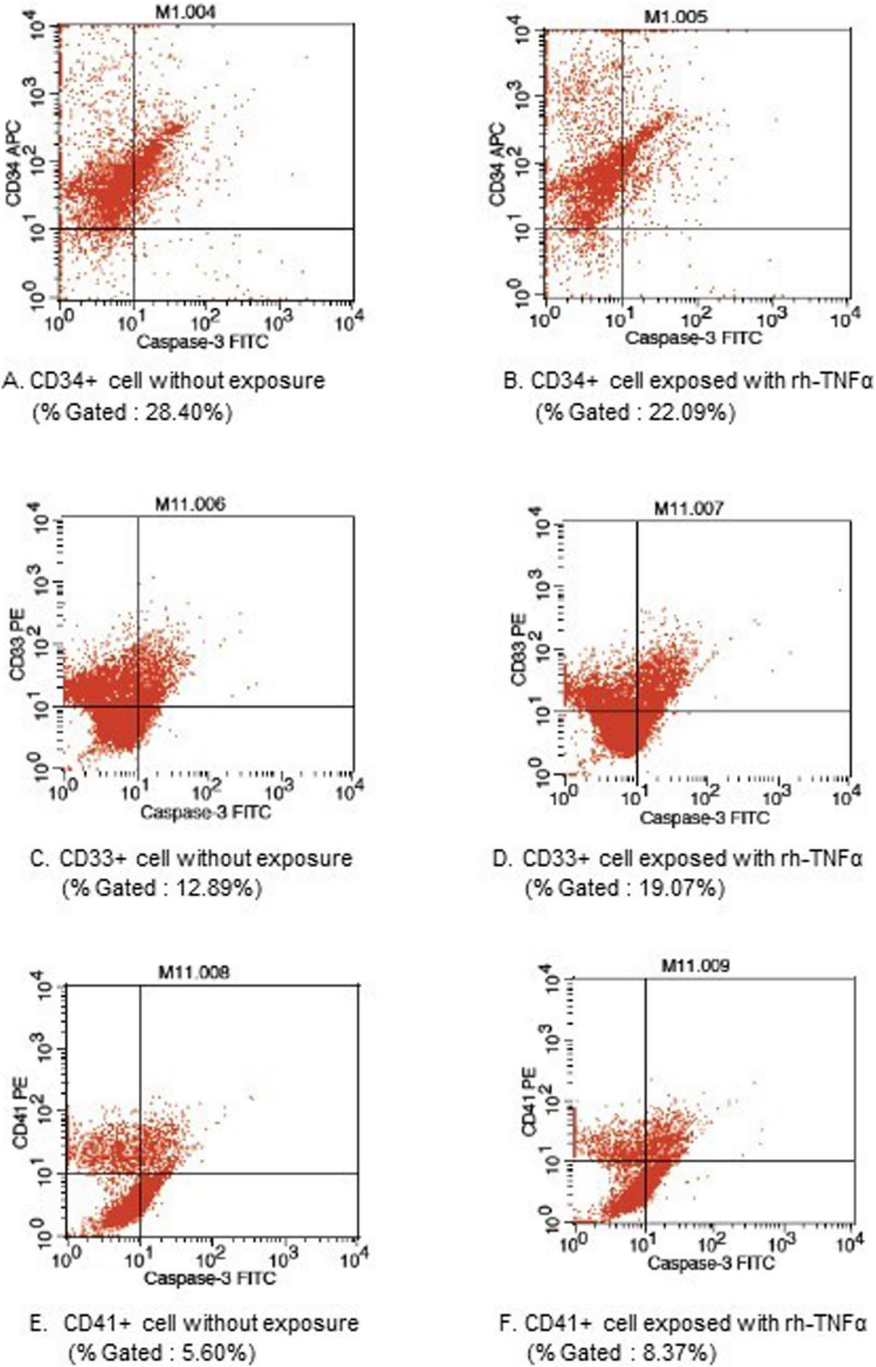
Caspase-3 Activity on MDS BMMC by Flowcytometry Analysis. A result of caspase-3 activity is indicated by gating in the upper right quadrant (UR). **A** CD34 + cell without exposure, **B** CD34 + cell exposed to rh-TNFα, **C** CD33 + cell without exposure, **D** CD33 + cell exposed to rh-TNFα, **E** CD41 + cell without exposure, **F** CD41 + cell exposed to rh-TNFα

The mean of Δ activity caspase-3 in CD33 + between MDS and control groups showed that there was a significant difference with p-value 0.006. The paired t test to analyze caspase activity in CD33 + without exposure and exposed to rhTNFα showed a significant difference in both groups, MDS with *p*-value < 0.001 and control with *p*-value 0.031. The paired t-test in CD41 + MDS without exposure and exposed to rhTNFα showed a significant difference with *p*-value 0.028. One of the results of caspase-3 activity shows in Fig. 3.

Due to the statistical significance in the one-way ANOVA test among the different cell group variants, we proceeded to conduct Tamhane’s post hoc test to identify which groups exhibit differences. The disparity in mean Δ caspase activity is negative (-), indicating that CD34 + caspase activity is lower than caspase-3 activity in its comparator progenitor cells, namely CD33 + and CD41 + cells. A significant difference in mean Δ caspase-3 activity was observed within the MDS group, specifically between CD34 + and CD33 + cells, as well as between CD34 + cells and CD41 + cells, with *p*-values of < 0.001 and 0.013, respectively.

## Discussion

### Exposure to rhTNFα induced apoptosis in MDS hematopoetic progenitor cells

The purpose of rhTNFα exposure is to induce these pro-apoptotic cytokines and observe the apoptotic response, specifically caspase-3 activity in CD34+, CD33+, and CD41 + MDS BMMC. Apoptosis may occur through either the intrinsic or extrinsic pathway. The TNFα apoptosis mechanism model operates through the extrinsic pathway.

The TNFα signaling pathway mechanism for initiating the apoptotic process begins with the binding of TNFα to TNFR-1 and TNFR-2 receptors. TNFR-1 mediates the cell death program by initially binding directly to the adapter protein TNF receptor-associated death domain (TRADD), then indirectly connecting to the FAS-associated protein with a death domain (FADD). This sequential process ultimately leads to the activation of the caspase cascade, culminating in the execution phase facilitated by caspase-3 [16–18].

Exposure of rhTNFα to hematopoietic progenitor cells is expected to result in the binding of rhTNFα to its receptor, TNFR1. This binding is anticipated to initiate cell death signals through the death-inducing signaling complex (DISC) and activate TRADD. Subsequently, caspase activation will be triggered, ultimately leading to the activation of caspase-3, which serves as the executor of apoptosis in hematopoietic progenitor cells in MDS, as shown in Fig. 1.

The role of TNFα in triggering apoptosis is supported by a research report that discovered a positive correlation between elevated serum TNFα levels and anemia in MDS patients. Furthermore, an increase in TNFα levels in the bone marrow was linked to heightened apoptosis during the early stages of MDS [19]. Several studies have also documented increased activity of caspase-1 and caspase-3 in hematopoiesis cells of MDS patients, with caspase-3 activity being ten times higher than that of caspase-1. Notably, the heightened caspase-3 activity is positively associated with increased TNFα levels [20, 21].

However, it is possible for caspase-3 activation to occur through interactions aside from rhTNFα exposure, although such instances were negligible in this study. We determined this by calculating the delta value (Δ) for Caspase-3 using the formula: the caspase-3 activity data after rhTNFα exposure subtracted by data before exposure (as the baseline). The positive and statistically significant delta values indicate that rhTNFa exposure can indeed increase caspase-3 activity.

The involvement of TNFα as an apoptosis trigger in MDS was confirmed by the elevated levels of TNFα in the serum of MDS patients. This increase in TNFα showed a positive correlation with clinical anemia, and an elevated rate of apoptosis in the early stages of MDS [19].

In MDS, there is an increasein the activity of both caspase-1 and caspase-3, with caspase-3 activity being ten times higher than caspase-1 [7, 13, 14]. This increased caspase-3 activity is associated with a high level of TNFα [20, 21]. Iriani et al. [22] discovered that the high levels of TNFα in MDS are initiated by an increase in the expression of TNFα mRNA, triggered by sCD40L stimulation.

A substantial portion of MDS cells undergo intramedullary apoptosis, which can be challenging to detect morphologically, as it predominantly occurs at an early stage. Therefore, the assessment of apoptosis in MDS should involve early markers of apoptosis, such as caspase-3.

### Apoptosis in CD34+

In Fig. 2A, the Δ caspase-3 value in the MDS group was negative, whereas it was positive, with a significant p-value. The Δ value was used to gauge the extent of the impact of rhTNFα exposure. This suggests that, in the MDS group, apoptosis had occurred in CD34 + cells even without exposure to rhTNFα, in contrast to the control group. This implies that CD34 + MDS cells naturally tend to undergo apoptosis.

Reports indicate that CD34 + MDS cells tend to undergo apoptosis, with their susceptibility to apoptosis being most pronounced among CD34 + progenitor cells. [11, 23] This susceptibility is attributed to defects in the progenitor stem cells. Due to these clonal abnormalities, daughter cells would develop into defective cells that are prone to apoptosis. Another study found that MDS CD34 + cells displayed higher apoptotic activity than normal cells, with the apoptosis rate in MDS CD34 + cells being 2.08 times faster than their proliferation rate [24]. In MDS, despite the bone marrow continuing to produce cells, the cell cycle is shorter. It is also known that over two-third of MDS cells at various stages of differentiation in the bone marrow undergo apoptosis.

### Apoptosis in CD33+

In Fig. 2B, the ∆ caspase-3 value in the MDS group was higher than that in the control group, with a significant p-value. This suggests that the exposure of rhTNFα led to increased apoptosis in both groups, but the impact of increasing apoptosis in CD33 + MDS cell group was more significant.

Raza et al. [11] found that in MDS, exposure of TNFα would result in increased apoptosis in both CD33 + and CD34 + cells. In MDS, despite the excess apoptosis in the bone marrow, granulocyte in the peripheral circulation tend to be more resistant to apoptosis. This occurs because sensitive granulocytes undergo apoptosis earlier in the bone marrow, while apoptosis-resistant granulocytes continue to enter the circulation. Apoptosis in the myeloid lineage is also affected by *myeloid-derived suppressor cells* (MDSCs). In MDS, there is an increase in circulating MDSCs. MDSCs induce apoptosis in hematopoietic progenitor cells and trigger the secretion of suppressive cytokines, IL10 and TGFβ, which suppress the development of immature myeloid cells [25].

### Apoptosis in CD41+

In Fig. 2C, caspase-3 activity in CD41 + in the MDS group exposed to rhTNFα was significantly higher compared to the group without exposure. This indicated that TNFα exposure to MDS megakaryocyte-platelet lineage cells leads to increased apoptosis. Cevic et al. [26], also observed that TNFα increased caspase-3 activity in platelets.

Apoptosis in megakaryocyte-platelets lineage of MDS can occur through the extrinsic pathway involving TNFα, but it predominantly takes place via the intrinsic pathway. This is supported by the finding of higher levels of Bax and Bak proteins [27], as well as the possibility of mitochondrial activation or the release of cytochrome C from the cytosol [28].

Following the maximum release of platelets (1000–3000 platelets), senescent megakaryocytes, characterized by a thin nucleus and cytoplasm, immediately undergo apoptosis and are phagocytosed by macrophages [29]. The apoptosis in MDS megakaryocytes occurs due to ineffective platelet production caused by the premature apoptosis of mature megakaryocytes in the intramedullary. Cytogenetic abnormalities in MDS clones primarily led to defects in megakaryocytes, manifesting in morphological abnormalities, and disturbances in nucleus differentiation and maturation. Moreover, these abnormalities are influenced by the MDS bone marrow microenvironment.

**Fig. 4:**
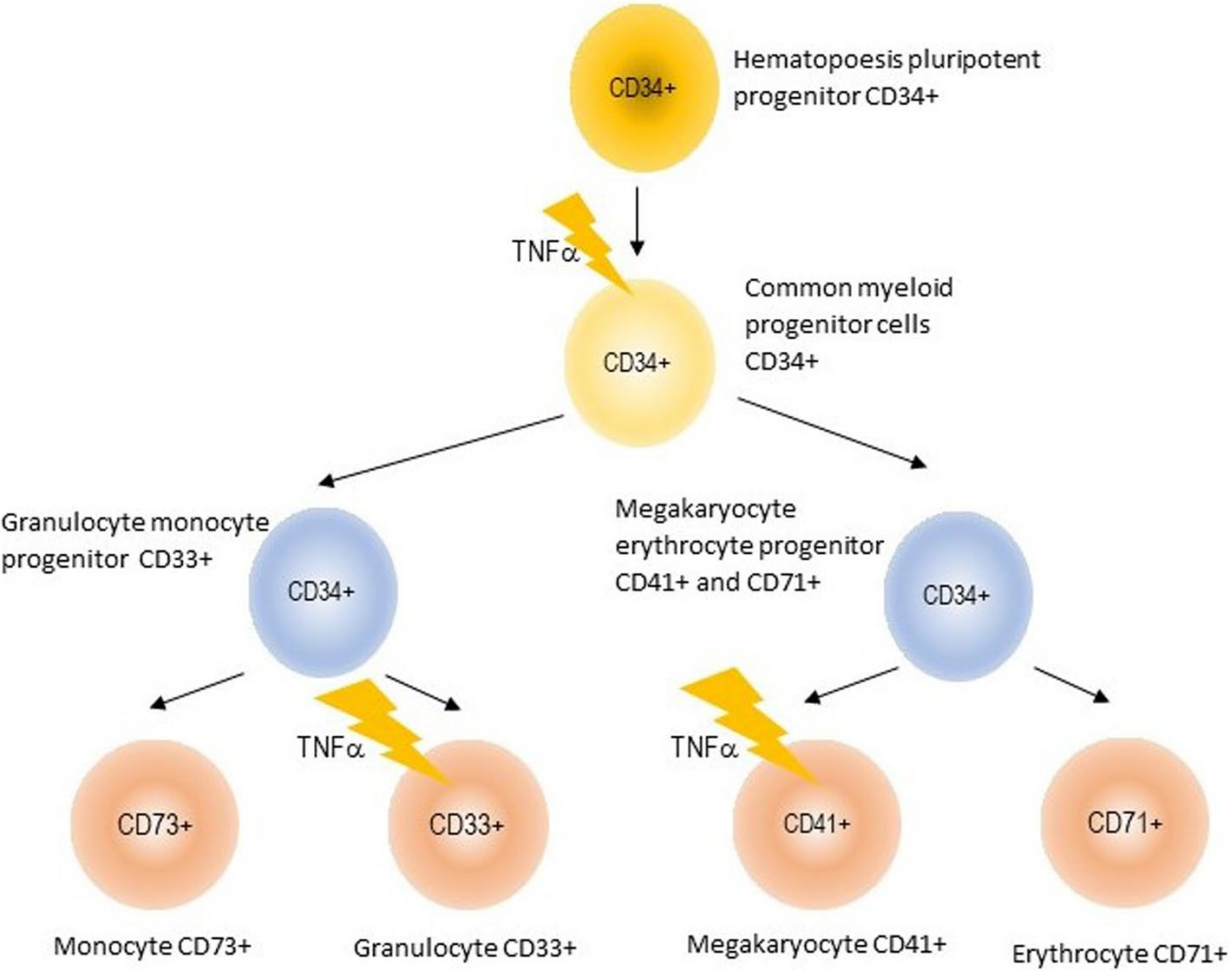
Scheme of Apoptosis Mechanism on MDS Hematopoesis Progenitor Cell by.  TNFα. Early hematopoesis progenitor cell express CD34 + surface marker. Then it will differentiate into specific myeloid progenitor (CD33+), megakaryocyte-thrombocyte progenitor (CD41+). This image illustrates that TNFα induces apoptosis in MDS hematopoesis progenitor cell, especially to cell that have differentiated into myeloid CD33 + and megakaryocyte-thrombocyte CD41+

To address the hypothesis regarding whether apoptosis in MDS progenitor cell occurs during the early stage or at a differentiation stage, a post hoc analysis was performed to examine the apoptotic activity of caspase-3 in CD34 + cells, representing early progenitors, and in CD33 + and CD41 + cells, representing differentiated progenitors. This study demonstrates that exposure to rhTNFα significantly increases caspase-3 activity, especially in hematopoietic progenitor cells that differentiate into the myeloid lineage CD33 + and the megakaryocyte-thrombocyte lineage CD41+, as opposed to early progenitor lineage CD34+, Fig. 4.

These findings shed light on why cytopenia in MDS is more pronounced than the increase in blast cells. Similar results were reported by Xia et al. [30], who examined apoptotic activity in MDS CD34+ (progenitor cells) and MDS CD34- (mature cells), and found higher apoptotic activity in mature cells. However, their study did not distinguish the specific stage of differentiation among mature cells.

These results contrast with the previous hypothesis, which suggested that apoptosis in MDS mainly occurs in early progenitor cells CD34+.

The reason MDS develops slowly but progressively lies in the fact that CD34 + MDS cells are long-term repopulating stem cells (LT-HSC) with a high self-renewal capacity but a slow proliferation rate. The MDS cell clone could persist for up to 17 years without clinically significant changes in bone marrow or peripheral blood. However, after prolonged perios, the bone marrow exhibits an 8.15% presence of dysplastic progenitor cells. These abnormal progenitor cells would significantly reduce the number of mature cells in the peripheral blood, leading to cytopenia [31].

This explanation helps clarify the typical phenomenon in MDS, where there is hypercellularity in the bone marrow but cytopenia in the peripheral blood. It suggests that apoptosis occurs mainly in differentiated progenitor cells rather than in early progenitor cells. Unlike other malignant cells that can further develop, MDS clonal cells fail to mature, leading to an accumulation of cells with increased apoptotic abilities.

## Conclusion

We have demonstrated that rhTNFα, a proapoptotic cytokine, plays a significant role in initiating apoptotic activity through caspase-3 in hematopoietic cells of MDS. This effect is particularly pronounced in cells that have already differentiated into myeloid lineage CD33 + and the megakaryocyte-thrombocyte lineage CD41+, as opposed to early progenitor cells CD34+. The results of this study have effectively substantiated the hypothesis that apoptosis predominantly takes place in differentiated progenitor cells, specifically in the myeloid and platelet lineages, as opposed to early progenitor cells in MDS hematopoiesis. This revised hypothesis differs from the initial one, which suggested that apoptosis mainly occurs in CD34 + early progenitor cells in MDS.

### Supplementary Information

**Additional file 1:** **Supplementary Table 1 . **Characteristics of The Study Subjects. **Supplementary Table 2. **Caspase-3 Activity in CD34+, CD33+ and CD41+ Cells Without exposure and exposure to rhTNFa. **Supplementary Table 3.   ***One Way* Anova test and Post Hoc test to D Caspase-3 in CD34+, CD33+ and CD41+ Cells.

## Data Availability

The datasets used and/or analyzed during the current study are available in our institutionalized database and the Copy Right of this study has been deposited in the repository of Yarsi University, or please contact the corresponding author upon reasonable request.
