# Supplementary material for: TNFα induces Caspase-3 activity in hematopoietic progenitor cells CD34+, CD33+, and CD41 + of myelodysplastic syndromes
Source: BMC Mol Cell Biol. 2023 Nov 21;24:33. doi: 10.1186/s12860-023-00495-0 (PMC10662645; doi:10.1186/s12860-023-00495-0)
Supplement: Supplementary file 1 — Additional file 1: Supplementary Table 1 . Characteristics of The Study Subjects. Supplementary Table 2. Caspase-3 Activity in CD34+, CD33+ and CD41+ Cells Without exposure and exposure to rhTNFa. Supplementary Table 3. One Way Anova test and Post Hoc test to D Caspase-3 in CD34+, CD33+ and CD41+ Cells. [file 12860_2023_495_MOESM1_ESM.docx]

**Supplementary Table 1 . Characteristics of The Study Subjects**

| **Case subjects** | **Bone Marrow Morphology** | **Cytogenetic Conclusion** | **Blas (%)** | **Hb**  **(g/dL)** | **Trombocyte (cells/µL)** | **Neutrophil**  **(cells/µL)** | **IPSS-R**  **(score)** |
| --- | --- | --- | --- | --- | --- | --- | --- |
| 1 | RCUD | Worse | 4 | 9,5 | 268.000 | 13.400 | High (5) |
| 2 | RCUD | Worse | 1,5 | 10,2 | 74.000 | 1.820 | Intermediate (4,5) |
| 3 | RCUD | Worse | 1.5 | 4,1 | 5.000 | 351 | Very high (7) |
| 4 | RCUD | No metaphase | 1,5 | 8,8 | 92.000 | 6.222 | NA |
| 5 | RCMD | Very worse | 3,5 | 10,2 | 58.000 | 3.996 | High (4,5) |
| 6 | RCMD | Good | 3.5 | 12.1 | 20.000 | 2.391 | Low (2,5) |
| 7 | RCMD | Intermediate | 4 | 5,6 | 111.000 | 5.175 | Intermediate (4,5) |
| 8 | RCMD | Good | 1 | 10,1 | 145.000 | 492 | Very low (1) |
| 9 | RCMD | **✝** | 1 | 5,5 | 38.000 | 225 | NA |
| 10 | RCMD | Good | 1,3 | 4,7 | 29.000 | 1.051 | Intermediate (3,5) |
| 11 | RCMD | No metaphase | 3,3 | 6,5 | 6.030 | 189.100 | NA |
| 12 | RAEB 1 | Very worse | 5 | 10,1 | 95.000 | 2.484 | Very high (7,5) |
| 13 | RAEB 1 | Good | 7 | 9,0 | 133.000 | 2.747 | Intermediate (4) |
| 14 | RAEB 1 | Good | 5,5 | 8,3 | 57.000 | 442 | Intermediate (4,5) |
| 15 | RAEB 1 | Intermediate | 6,5 | 9,5 | 44.000 | 6.443 | High (6) |

RCUD= refracrory cytopenias with unilineage displasia, RCMD= refractory anemia with multilineage dysplasia, RAEB-1= refractory anemia with excess blast-1, NA= not applicable, IPSS-R = International Prognostic Scoring System-Revised WHO 2008.

**Supplementary Table 2. Caspase-3 Activity in CD34+, CD33+ and CD41+ Cells Without exposure and exposure to rhTNFα**

| **Caspase-3 activity (%)** | | | |
| --- | --- | --- | --- |
| **Cells** | **MDS**  **(n=15)** | **Control**  **(n=8)** | **p value*** |
| CD34+ |  |  |  |
| Untreated | 22,67 ± 10,75 | 18,84 ± 8,79 | 0,664 |
| Treated rhTNFα | 19,53 ± 7,58 | 23,72 ± 10,12 | 0,212 |
| Δ Caspase 3 | -3,13 ± 5,18 | 4,87 ± 6,73 | **0,016** |
| P value ** | **0,034** | **0,008** |  |
| CD33+ |  |  |  |
| Untreated | 26,03 ± 17,82 | 46,86 ± 29,00 | 0,094 |
| Treated rhTNFα | 34,59 ± 18,95 | 49,961 ± 30,05 | 0,218 |
| Δ Caspase 3 | 8,56 ± 5,29 | 3,10 ± 3,25 | **0,006** |
| P value ** | **< 0,001** | **0,031** |  |
| CD41+ |  |  |  |
| Untreated | 8,49 ± 8,09 | 11,39 ± 8,84 | 0,454 |
| Treated rhTNFα | 11,28 ± 10,87# | 12,87 ± 8,19 | 0,698 |
| Δ Caspase 3 | 2,79 ± 4.42# | 1,48 ± 6,75 | 0,631 |
| P value ** | **0,028** | 0,553 |  |

Mean (SD), Delta (Δ ) caspase-3 activity is the difference between caspase-3 activity of BMMC exposed to rhTNFα with no exposure,

*Independent sample t test

** Paired sample t test

**Supplementary Table 3. *One Way* Anova test and Post Hoc test to** Δ **Caspase-3 in CD34+, CD33+ and CD41+ Cells**

| **Cells** | **One Way Anova** | | |  | **Post Hoc** | |
| --- | --- | --- | --- | --- | --- | --- |
|  |  | **Δ Caspase-3 %** | **p value** |  |  | **p value** |
| **MDS** | CD34+ | -3,13 (5,18) | **< 0,001** |  | CD34+ vs CD33+ | **<0,001** |
|  | CD33+ | 8,56 (5,29) |  |  | CD34+ vs CD41+ | **0,013** |
|  | CD41+ | 2,79 (4,42) |  |  |  |  |
| **Control** | CD34+ | 4,87 (6,73) | 0,809 |  |  |  |
|  | CD33+ | 3,10 (3,25) |  |  |  |  |
|  | CD41+ | 1,48 (6,75) |  |  |  |  |

Mean (SD)
